# Supplementary material for: Bat coronavirus phylogeography in the Western Indian Ocean
Source: Sci Rep. 2020 Apr 23;10:6873. doi: 10.1038/s41598-020-63799-7 (PMC7181612; doi:10.1038/s41598-020-63799-7)
Supplement: Supplementary file 1 — Supplementary information. [file 41598_2020_63799_MOESM1_ESM.pdf]

Supplementary information: Bat coronavirus phylogeography in the Western Indian Ocean

Léa Joffrin, Steven M. Goodman, David A. Wilkinson, Beza Ramasindrazana, Erwan Lagadec, Yann Gomard, Gildas Le Minter, Andréa Dos Santos, M. Corrie Schoeman, Rajendraprasad Sookhareea, Pablo Tortosa, Simon Julienne, Eduardo S. Gudo, Patrick Mavingui and Camille Lebarbenchon

Research permits for the collection of biological materials

Reunion Island

Samples were collected as part of a previous study on lyssavirus infection in bats in the Indian Ocean (1), under a research permit delivered by the Préfecture de La Réunion: "Arrêté préfectoral du 11 Février 2013 and Arrêté préfectoral du 11 Septembre 2014 (N°2014-07)".

Mauritius

Samples were collected as part of a previous study on lyssavirus infection in bats in the Indian Ocean (1), under a memorandum of agreement for the supply of biological material by Government of Mauritius (delivered by the National Park and Conservation Service for authorization of Mauritius), signed 17 December 2010 and 09 January 2013. CITES permit from the Mauritian national authority was issued for tissue export (permit MU120933) to Reunion Island.

Mayotte

Samples were collected as part of a previous study on lyssavirus infection in bats in the Indian Ocean (1), under a research permit delivered by the "Préfecture de Mayotte: Arrêté N°158/DEAL/SEPR/2014".

Madagascar

Samples were collected as part of previous studies on infectious agents in Malagasy wildlife (1–4), under the following research permits delivered by the "Direction du Système des Aires Protégées and Direction Générale de l'Environnement et des Forêts"; Madagascar National Parks: 350/10/MEF/SG/DGF/DCB.SAP/SCB, 032/12/MEF/SG/DGF/ DCB.SAP/SCBSE, 067/12/MEF/SG/DGF/DCB.SAP/SCBSE, 194/12/ MEF/SG/DGF/DCB.SAP/SCB, N◦283/11/MEF/SG/DGF/DCB.SAP/SCB, N◦077/12/MEF/SG/DGF/DCB.SAP/SCBSE, 238/14/MEEF/SG/ DGF/DCB.SAP/SCB and 268/14/MEEF/SG/DGF/DCB.SAP/SCB. A CITES permit from the Malagasy national authority was issued for tissue export (permit 243C-EA06/MG12) to Reunion island.

Seychelles (Mahe)

Samples were collected as part of a previous study on lyssavirus infection in bats in the Indian Ocean (1), under the following research permit delivered by the Direction of Wildlife, Trade and Conservation Section and the Ministry of Environment and Energy of Republic of Seychelles: Export permits Agreement of 5 March 2014. CITES permit from the Republic of Seychelles was issued for export (permit N°1772) to Reunion Island.

Mozambique (Inhassoro district, Inhambane province)

Samples were collected as part of a previous study on astrovirus infection in bats in the Indian Ocean (5), under a research permit delivered by the Ministry of Health (N°S/N/SDI/0233/15).

Type of collected samples

Organs were collected before 2014, stored in liquid nitrogen in the field and then at −80°C upon arrival at the laboratory (Appendix Table 1). From 2014 onward, feces, rectal, and oral swabs were collected (Appendix Table 1) and placed in 1mL brain heart infusion medium (Conda, Madrid, Spain) supplemented with penicillin G (1,000 units/mL), streptomycin (1 mg/mL), kanamycin (0.5 mg/mL), gentamicin (0.25 mg/mL) and amphotericin B (0.025 mg/mL). These samples were also stored in liquid nitrogen in the field and at −80°C upon arrival at the laboratory (Appendix Table 1).

REFERENCES

1. Mélade J, McCulloch S, Ramasindrazana B, Lagadec E, Turpin M, Pascalis H, et al. Serological Evidence of Lyssaviruses among Bats on Southwestern Indian Ocean Islands. PLoS One. 2016;11(8):e0160553.
2. Wilkinson DA, Mélade J, Dietrich M, Ramasindrazana B, Soarimalala V, Lagadec E, et al. Highly Diverse Morbillivirus-Related Paramyxoviruses in Wild Fauna of the Southwestern Indian Ocean Islands: Evidence of Exchange between Introduced and Endemic Small Mammals. J Virol. 2014;88(15):8268–77.
3. Gomard Y, Dietrich M, Wieseke N, Ramasindrazana B, Lagadec E, Goodman SM, et al. Malagasy bats shelter a considerable genetic diversity of pathogenic Leptospira suggesting notable host-specificity patterns. FEMS Microbiol Ecol. 2016;92(4):fiw037.
4. Mélade J, Wieseke N, Ramasindrazana B, Flores O, Lagadec E, Gomard Y, et al. An eco-epidemiological study of Morbilli-related paramyxovirus infection in Madagascar bats reveals host-switching as the dominant macro-evolutionary mechanism. Sci Rep. 2016;12(6):23752.
5. Hoarau F, Minter G Le, Joffrin L, Schoeman MC, Lagadec E, Ramasindrazana B, et al. Bat Astrovirus in Mozambique. Virol J. 2018;15(104):1–5.

Supplementary tables

Table S1. Number of positive samples and sequences generated with the Real-Time PCR detection system, and the classic PCR system used to generate sequences for phylogenetic analyses.

				Detection system			Phylogeny system	
**Location**	**Sampling year**	**Bat family**	**Bat species**	**N tested**	**N detected**	**N sequences**	**N tested**	**N sequences**
**Madagascar**	**2012**	**Molossidae**	*Mops midas*	21	4	4	4	0
		**Rhinonycteridae**	*Triaenops menamena*	21	2	0	2	0
	**2013**	**Molossidae**	*Mormopterus jugularis*	63	10	7	10	5
		**Pteropodidae**	*Rousettus madagascariensis*	45	6	6	6	2
	**2014**	**Rhinonycteridae**	*Triaenops menamena*	13	2	2	2	0
**Mayotte**	**2014**	**Molossidae**	*Chaerephon sp*	4	1	1	1	1
			Chaerephon pusillus	61	7	7	7	5
**Mozambique**	**2015**	**Hipposideridae**	*Hipposideros caffer*	59	10	6	10	3
		**Miniopteridae**	*Miniopterus mossambicus*	21	4	4	4	4
		**Molossidae**	*Mops condylurus*	54	11	11	11	4
		**Nycteridae**	*Nycteris thebaica*	14	4	3	4	4
		**Rhinolophidae**	*Rhinolophus lobatus*	9	6	6	6	4
			Rhinolophus rhodesiae	30	9	9	9	9
			Rhinolophus sp	2	1	1	1	1
		**Rhinonycteridae**	*Triaenops afer*	51	9	8	9	8
**Reunion**	**2015**	**Molossidae**	*Mormopterus francoismoutoui*	50	2	2	2	1
					88	77	88	51

Table S2. Nucleotide sequence similarity obtained with BLAST.

ID	species	% identity	GENBANK Sequence	Accession GENBANK ID
MAY004	*Chaerephon pusillus*	92%	Chaerephon bat coronavirus/Kenya/KY22/2006 polyprotein (ORF1ab) gene	HQ728486
MAY015	*Chaerephon pusillus*	92%	Chaerephon bat coronavirus/Kenya/KY22/2006 polyprotein (ORF1ab) gene	HQ728486
MAY025	*Chaerephon pusillus*	92%	Chaerephon bat coronavirus/Kenya/KY22/2006 polyprotein (ORF1ab) gene	HQ728486
MAY027	*Chaerephon pusillus*	92%	Chaerephon bat coronavirus/Kenya/KY22/2006 polyprotein (ORF1ab) gene	HQ728486
MAY033	*Chaerephon pusillus*	92%	Chaerephon bat coronavirus/Kenya/KY22/2006 polyprotein (ORF1ab) gene	HQ728486
MAY051	*Chaerephon sp*	92%	Chaerephon bat coronavirus/Kenya/KY22/2006 polyprotein (ORF1ab) gene	HQ728486
RB369	*Mormopterus francoismoutoui*	90%	Chaerephon bat coronavirus/Kenya/KY22/2006 polyprotein (ORF1ab) gene	HQ728486
SMG18218	*Mormopterus jugularis*	90%	Chaerephon bat coronavirus/Kenya/KY22/2006 polyprotein (ORF1ab) gene	HQ728486
SMG18223	*Mormopterus jugularis*	90%	Chaerephon bat coronavirus/Kenya/KY22/2006 polyprotein (ORF1ab) gene	HQ728486
SMG18225	*Mormopterus jugularis*	90%	Chaerephon bat coronavirus/Kenya/KY22/2006 polyprotein (ORF1ab) gene	HQ728486
SMG18231	*Mormopterus jugularis*	90%	Chaerephon bat coronavirus/Kenya/KY22/2006 polyprotein (ORF1ab) gene	HQ728486
SMG18238	*Mormopterus jugularis*	90%	Chaerephon bat coronavirus/Kenya/KY22/2006 polyprotein (ORF1ab) gene	HQ728486
SMG18866	*Rousettus madagascariensis*	98%	Kenya bat coronavirus BtKY89 RNA-dependent RNA polymerase gene, partial cds	GU065433

SMG18930	*Rousettus madagascariensis*	98%	Kenya bat coronavirus BtKY89 RNA-dependent RNA polymerase gene, partial cds	GU065433
SMG18951	*Hipposideros caffer*	96%	229E-related bat coronavirus strain BtKY229E-8	KY073748
SMG18958	*Triaenops afer*	98%	NL63-related bat coronavirus strain BtKYNL63-15, complete genome	KY073746
SMG18970	*Nycteris thebaica*	88%	Betacoronavirus BtCoV/KW2E-F93/Nyc_spec/GHA/2010 RNA-dependent RNA polymerase gene, partial cds; and spike glycoprotein gene, complete cds	JX899383
SMG18971	*Nycteris thebaica*	88%	Betacoronavirus BtCoV/KW2E-F93/Nyc_spec/GHA/2010 RNA-dependent RNA polymerase gene, partial cds; and spike glycoprotein gene, complete cds	JX899383
SMG18972	*Nycteris thebaica*	88%	Betacoronavirus BtCoV/KW2E-F93/Nyc_spec/GHA/2010 RNA-dependent RNA polymerase gene, partial cds; and spike glycoprotein gene, complete cds	JX899383
SMG18987	*Hipposideros caffer*	96%	229E-related bat coronavirus strain BtKY229E-8, complete genome	KY073748
SMG18990	*Rhinolophus lobatus*	87%	Kenya bat coronavirus BtKY83 RNA-dependent RNA polymerase gene, partial cds	GU065427
SMG18991	*Rhinolophus lobatus*	87%	Kenya bat coronavirus BtKY83 RNA-dependent RNA polymerase gene, partial cds	GU065427
SMG18992	*Rhinolophus lobatus*	87%	Kenya bat coronavirus BtKY83 RNA-dependent RNA polymerase gene, partial cds	GU065427
SMG18993	*Triaenops afer*	98%	NL63-related bat coronavirus strain BtKYNL63-15, complete genome	KY073746
SMG18996	*Triaenops afer*	98%	NL63-related bat coronavirus strain BtKYNL63-15, complete genome	KY073746
SMG18997	*Triaenops afer*	98%	NL63-related bat coronavirus strain BtKYNL63-15, complete genome	KY073746
SMG19002	*Hipposideros caffer*	96%	229E-related bat coronavirus strain BtKY229E-8, complete genome	KY073748

SMG19004	*Miniopterus mossambicus*	98%	Miniopterus bat coronavirus/Kenya/KY27/2006 polyprotein (ORF1ab) gene	HQ728484
SMG19006	*Miniopterus mossambicus*	97%	Miniopterus bat coronavirus/Kenya/KY27/2006 polyprotein (ORF1ab) gene	HQ728484
SMG19013	*Miniopterus mossambicus*	98%	Miniopterus bat coronavirus/Kenya/KY27/2006 polyprotein (ORF1ab) gene	HQ728484
SMG19015	*Miniopterus mossambicus*	98%	Miniopterus bat coronavirus/Kenya/KY27/2006 polyprotein (ORF1ab) gene	HQ728484
SMG19020	*Rhinolophus rhodesiae*	87%	Kenya bat coronavirus BtKY83 RNA-dependent RNA polymerase gene, partial cds	GU065427
SMG19024	*Rhinolophus rhodesiae*	98%	Miniopterus bat coronavirus/Kenya/KY27/2006 polyprotein (ORF1ab) gene	HQ728484
SMG19026	*Rhinolophus sp*	87%	Kenya bat coronavirus BtKY83 RNA-dependent RNA polymerase gene, partial cds	GU065427
SMG19027	*Rhinolophus rhodesiae*	87%	Kenya bat coronavirus BtKY83 RNA-dependent RNA polymerase gene, partial cds	GU065427
SMG19028	*Rhinolophus rhodesiae*	87%	Kenya bat coronavirus BtKY83 RNA-dependent RNA polymerase gene, partial cds	GU065427
SMG19029	*Rhinolophus lobatus*	82%	Rhinolophus bat coronavirus BtCoV/4307-2 RNA-dependent RNA polymerase (RdRp) gene, partial cds	KP876528
SMG19030	*Rhinolophus rhodesiae*	87%	Kenya bat coronavirus BtKY83 RNA-dependent RNA polymerase gene, partial cds	GU065427
SMG19031	*Rhinolophus rhodesiae*	87%	Kenya bat coronavirus BtKY83 RNA-dependent RNA polymerase gene, partial cds	GU065427
SMG19032	*Rhinolophus rhodesiae*	87%	Kenya bat coronavirus BtKY83 RNA-dependent RNA polymerase gene, partial cds	GU065427
SMG19037	*Rhinolophus rhodesiae*	87%	Kenya bat coronavirus BtKY83 RNA-dependent RNA polymerase gene, partial cds	GU065427
SMG19038	*Rhinolophus rhodesiae*	82%	Rhinolophus bat coronavirus BtCoV/4307-2 RNA-dependent RNA polymerase (RdRp) gene, partial cds	KP876528
SMG19141	*Mops condylurus*	98%	Chaerephon bat coronavirus/Kenya/KY22/2006 polyprotein (ORF1ab) gene	HQ728486

SMG19154	*Triaenops afer*	98%	NL63-related bat coronavirus strain BtKYNL63-15, complete genome	KY073746
SMG19174	*Triaenops afer*	98%	NL63-related bat coronavirus strain BtKYNL63-15, complete genome	KY073746
SMG19183	*Triaenops afer*	98%	NL63-related bat coronavirus strain BtKYNL63-15, complete genome	KY073746
SMG19185	*Triaenops afer*	98%	NL63-related bat coronavirus strain BtKYNL63-9a	KY073744
SMG19207	*Mops condylurus*	98%	Chaerephon bat coronavirus/Kenya/KY22/2006 polyprotein (ORF1ab) gene	HQ728486
SMG19210	*Mops condylurus*	98%	Chaerephon bat coronavirus/Kenya/KY22/2006 polyprotein (ORF1ab) gene	HQ728486
SMG19217	*Mops condylurus*	98%	Chaerephon bat coronavirus/Kenya/KY22/2006 polyprotein (ORF1ab) gene	HQ728486

Table S3. Number of tested and positive samples, per location, bat family, species, samples types, and collection year.

					N tested per type of samples				N positive (%)			
					Organs		Feces	Swabs	Organs		Feces	Swabs
Location	Bat family	Bat species	Collection Year	N tested	Lung	Intestine			Lung	Intestine		
Madagascar	Hipposideridae	*Macronycteris commersoni*	2012	19		19						
	Miniopteridae	*Miniopterus manavi*	2012	19		19						
		Miniopterus gleni	2012	14		14						
			2014	2				2				
		Miniopterus griveaudi	2014	28				28				
		Miniopterus mahafaliensis	2012	8		8						
		Miniopterus sororculus	2012	8		8						
	Molossidae	*Chaerephon atsinanana*	2013	29		29						
		Chaerephon leucogaster	2018	45				45				
		Mops leucostigma	2018	94				94				
		Mops midas	2012	19		19				4 (21.1)		
			2018	2				2				
		Mormopterus jugularis	2013	22		22				10 (45.5)		
			2014	41				41				
		Otomops madagascariensis	2012	12		12						
			2014	6				6				
	Pteropodidae	***Rousettus madagascariensis***	2014	45				45				6 (13.3)
	Rhinonycteridae	*Paratriaenops furculus*	2014	32				32				
		Triaenops menamena	2012	21		21				2 (9.5)		
			2014	13				13				2 (15.4)
	Vespertilionidae	*Myotis goudoti*	2014	17				17				
		Neoromicia malagasyensis	2013	2		2						

		Neoromicia matroka	2013	4		4						
		Pipistrellus hesperidus	2014	5				5				
Mauritius	Molossidae	*Momopterus acetabulosus*	2012	6		6						
	Pteropodidae	*Pteropus niger*	2012	48	48							
	Emballonuridae	*Taphosius mauritianus*	2014	2				2				
Mayotte	Molossidae	***Chaerephon sp***	2014	4			4				1 (25.0)	
		Chaerephon pusillus	2014	60			60				7 (11.6)	
	Pteropodidae	*Pteropus seychellensis comorensis*	2014	22				22				
Mozambique	Hipposideridae	***Hipposideros caffer***	2015	59				59				10 (16.9)
	Miniopteridae	***Miniopterus mossambicus***	2015	21				21				4 (19.0)
	Molossidae	***Mops condylurus***	2015	54				54				11 (20.4)
	Nycteridae	***Nycteris thebaica***	2015	14				14				4 (28.6)
	Rhinolophidae	***Rhinolophus lobatus***	2015	9				9				6 (66.7)
		Rhinolophus mossambicus	2015	20				20				
		Rhinolophus rhodesiae	2015	30				30				9 (30.0)
		Rhinolophus sp	2015	2				2				1 (50.0)
	Rhinonycteridae	***Triaenops afer***	2015	51				51				9 (17.7)
	Vespertilionidae	*Neoromicia nana*	2015	2				2				
		Scotophilus viridis	2015	2				2				
Reunion	Molossidae	***Mormopterus francoismoutoui***	2015	50				50				2 (4.0)

Seychelles	Pteropodidae	*Pteropus seychellensis seychellensis*	2014	50	50						
		TOTAL		**1013**	98	183	64	668	16	8	64

Table S4. Cytochrome *b* gene sequences accession numbers from western Indian Ocean bats. *: sequences generated in this study.

Bat family	Bat species	Cytb GenBank accession ID
Hipposideridae	*Hipposideros caffer*	FJ347980
Miniopteridae	*Miniopterus mossambicus*	MN273501*
Molossidae	*Chaerephon pusillus*	GQ489154
	Mops condylurus	GQ489181
	Mormopterus francoismoutoui	MN273499*
	Mormopterus jugularis	KR606332
Nycteridae	*Nycteris thebaica*	MN273500*
Pteropodidae	*Rousettus madagascariensis*	GU228727
Rhinolophidae	*Rhinolophus lobatus*	MN273497*
	Rhinolophus rhodesiae	MN273498*
Rhinonycteridae	*Triaenops afer*	EU798748

Supplementary figures

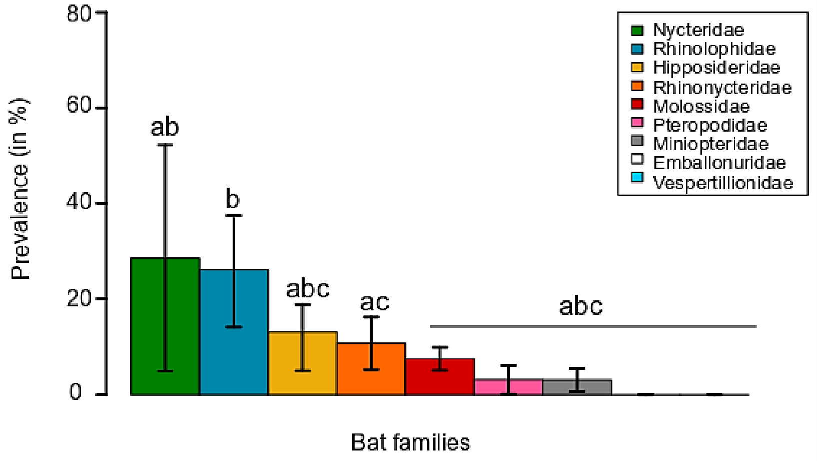

Figure S1. Mean CoV prevalence (± 95% confidence interval) as function of the bat family. Letters a–c above the bars refer to significantly different averages based upon a Pairwise test. Bars can have more than one letter to reflect the "overlap" between them.

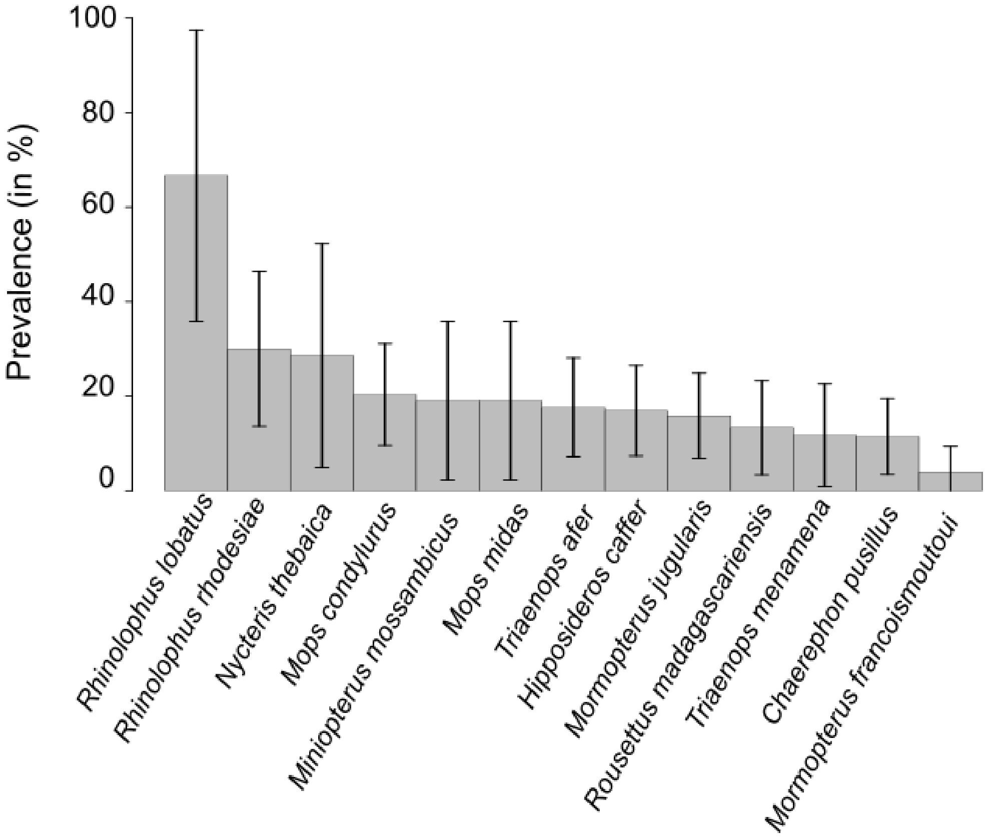

Figure S2. Mean CoV prevalence (mean ± 95% confidence interval) as function of the bat species.

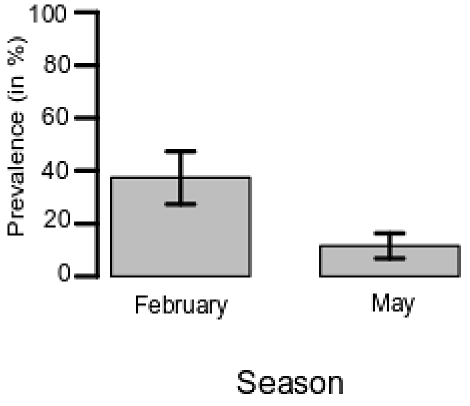

Figure S3. Mean CoV prevalence (mean ± 95% confidence interval) as function of the bat sampling season in Mozambique.
